# Enhancing patient outcomes: the role of clinical utility in guiding healthcare providers in curating radiology AI applications

**DOI:** 10.3389/fdgth.2024.1359383

**Published:** 2024-03-07

**Authors:** Franziska Lobig, Jacob Graham, Apeksha Damania, Brian Sattin, Joana Reis, Prateek Bharadwaj

**Affiliations:** ^1^ Digital & Commercial Innovation, Pharmaceuticals, MACS Radiology, Bayer AG, Berlin, Germany; ^2^ Life Sciences, Guidehouse Inc, New York, NY, United States

**Keywords:** radiology, imaging diagnostics, artificial intelligence, clinical utility, digital health

## Abstract

With advancements in artificial intelligence (AI) dominating the headlines, diagnostic imaging radiology is no exception to the accelerating role that AI is playing in today's technology landscape. The number of AI-driven radiology diagnostic imaging applications (digital diagnostics) that are both commercially available and in-development is rapidly expanding as are the potential benefits these tools can deliver for patients and providers alike. Healthcare providers seeking to harness the potential benefits of digital diagnostics may consider evaluating these tools and their corresponding use cases in a systematic and structured manner to ensure optimal capital deployment, resource utilization, and, ultimately, patient outcomes—or clinical utility. We propose several guiding themes when using clinical utility to curate digital diagnostics.

## Introduction

Recent advances in artificial intelligence (AI) have resulted in a rise in AI-driven radiology diagnostic imaging applications (digital diagnostics). These applications can detect, quantify, and classify radiological anomalies, resulting in a range of benefits such as improved accuracy, reduced inter/intra-observer variability, and the ability to automate/quantify areas of interest (1–3). More than 12,000 papers describing the use of AI in healthcare were published in 2019 (4), and by the end of 2022, almost 300 digital diagnostic applications were approved by the Food and Drug Administration (FDA) or CE-marked (5, 6). However, with no global central information repository, the real number of regulated applications is likely to be much higher.

Nevertheless, AI applications face a number of barriers to adoption in the healthcare space. In contrast to other industries, the level of scientific rigor required for broad adoption is high (7), and the business model and buying process to enable scalable reimbursement is still taking shape (8). With hospitals recovering from the aftermath of COVID-19 and reimbursement models moving towards value-based care in the US, digital diagnostics have the potential to transform healthcare. These tools can create value by supporting care pathway standardization, workflow efficiency enhancement, treatment decision support, and ultimately, care quality and outcomes improvement. However, as the number of digital diagnostics increases, the selection of these solutions can become overwhelming for radiology departments, treatment specialists, and hospital administrators. While there are several aspects to consider when evaluating digital diagnostics (interoperability, risk of bias, etc.), the potential for demonstrating clinical utility is a useful dimension for understanding a technology's ability to impact patient outcomes.

The concept of clinical utility is often based on the premise of improving net health outcomes by influencing patient management and treatment decisions. Clinical utility has been used by payers and health technology assessment (HTA) authorities for years to adjudicate the value of genetic lab-based testing. The concept of clinical utility may be applicable to digital diagnostics and could be used by providers to evaluate and prioritize AI investments. In this paper, we propose 4 guiding themes for healthcare providers to consider when curating digital diagnostics on the basis of clinical utility:

(1) Does the application address the unmet needs that underpin the clinical burden of disease?
(2) Do the performance and features of the AI application address the range of unmet needs associated with standard of care?
(3) Does the level of documented evidence demonstrate a credible impact on clinical decision-making and patient outcomes?
(4) What is the practice economic impact of the AI Application?

## Digital diagnostic landscape

Digital diagnostics are being developed across many different indications, imaging modalities, and use cases. To better understand the current role of digital diagnostics in healthcare, a search of available FDA-cleared or CE-marked AI applications was conducted in June 2022 with databases from the American College of Radiology's (ACR) Data Science Institute® and the Department of Medical Imaging at the Radboud University Medical Center's Diagnostic Image Analysis Group: AI for Radiology (5, 6). In total, 294 applications across 160 developers were identified. The applications were largely concentrated across 6 therapeutic areas (cardiology, oncology, neurology, hepatology, musculoskeletal, and respiratory/pulmonology) and 6 modalities (computed tomography [CT], x-ray, magnetic resonance [MR] spectroscopy, magnetic resonance imaging [MRI], ultrasound [US], and positron emission tomography [PET]).

Upon review of the applications, a handful of common use cases emerged based on the intended impact of the digital diagnostics on patient management and outcomes. We noted that a little less than half of the applications were designed to improve workflow efficiencies via radiology workflow automation, enhanced anatomical visualization, and automated reporting and worklist prioritization. While workflow efficiency applications create operational value, they need not necessarily lead to better patient outcomes, and therefore, would not satisfy the criteria for clinical utility.

Upon further review of these clinical applications, a common set of use cases emerge based on the role of the technology in patient management (Table 1): (1) earlier or more precise diagnosis, (2) patient triaging, (3) treatment decision optimization, (4) avoiding invasive and/or risky diagnostic procedures, and (5) predicting the risk of developing a disease or condition. While the use cases are not mutually exclusive, it is common for digital diagnostic developers to initially focus on a single use case for purposes of validation and evidence generation.

**Table 1:** Digital diagnostic use case examples.

Category	Use case	Examples
Clinical impact	Earlier or more precise diagnosis	• Algorithm to identify vertebral compression fractures (VCF) and low bone mineral density (BMD) from routine abdomen CT scans for early diagnosis of osteoporosis • Automated normative quantitative assessment of brain MRI for early identification of atrophy and Alzheimer's disease
	Patient triaging	• AI-based solution that analyzes CT images and flags incidental pulmonary embolisms (PE) (elective or emergency scans) to facilitate triage of patients in need of care coordination and access to catheter-directed interventional therapy thus reducing PE mortality and hospital stay duration • Application to automatically identify suspected large vessel occlusion strokes on CT angiogram and alert the on-call stroke team within minutes thus improving time-to-treatment and reducing hospital stay duration • Software to automatically identify regions of the brain and generate scores to help physicians quickly assess patient eligibility for thrombectomy
	Optimizing treatment decisions	• Software to evaluate liver health and individual Couinaud segments to identify patients at risk of poor post-surgical outcomes and longer hospital stay after liver resection surgery • Software to automatically identify regions of the brain and generate scores to help physicians quickly assess patient eligibility for thrombectomy • Algorithm to analyze various physical attributes of a renal stone on non-contrast enhanced CT scan slices that are relevant in predicting stone-free rates and the outcome of lithotripsy procedure performed on the stone
	Avoiding invasive and/or risky diagnostic procedures	• Software for non-invasive quantification of coronary obstruction to reduce the need for invasive coronary angiogram • Tool for non-invasive quantification of liver parameters to reduce the need for liver biopsy
	Predicting the risk of developing a disease or condition	• Software to identify and risk stratify incidentally detected pulmonary nodules (IPNs) into low- or high-risk categories via a lung cancer prediction score to help clinicians track at-risk patients and make optimal clinical management decisions • Algorithm to automatically detect coronary artery calcification (CAC) from pre-exisiting chest CT scans, to help identify patients at high risk of coronary artery disease • Algorithm that computes a trabecular bone score (TBS), which provides clinical information on the microarchitecture of the bone to identify patients at a high risk of fractures
Workflow efficiency	Improving operational workflows and generating cost efficiencies^a^	• Application that performs automated image segmentation/reconstruction to improve orthopedic surgical planning and reduce total O.R. time • Algorithm that identifies chest x-rays with no abnormalities, automating high-confidence healthy patient reports, reducing radiologist workload • Algorithm to visualize, quantify, and analyze complex flow patterns on 4D flow cardiovascular magnetic resonance imaging resulting in a 30% reduction in scan time

^a^ Applications under this use case category on their own are not intended to meet the criteria of clinical utility.

## Theme 1: does the application address the unmet needs that underpin the clinical burden of disease?

When curating diagnostics based on clinical utility, it is important to first prioritize clinical unmet needs based on their ability to be addressed with a digital solution.

Key questions to consider:

• What patient populations have disproportionally worse outcomes than others?
• What is the clinical burden of disease in terms of scope and severity, incidence/prevalence, disease duration, and time sensitivity of treatment?
• What patient outcomes are most negatively impacted (clinical, functional, quality of life, economic)?
• What role, if at all, do current diagnostics and treatments have on patient outcomes (e.g., mis-diagnosis rates, level of invasiveness, complication/adverse event rates)
• How does imaging play a role in treatment decision-making amongst other biomarkers and non-clinical factors (insurance, patient choice, etc.)? What is the duration of time between imaging, treatment selection, and patient outcomes?

Epidemiology data can be helpful for prioritizing which diseases to focus on, based on the size and significance of the problem to be addressed. Metrics such as mortality rates, complication rates, disease adjusted life years (DALYs), and average discharge costs can help with prioritizing indications based on the severity of the condition, while metrics such as incidence/prevalence, admission rates, and diagnosis rates can establish how frequent the condition presents within a hospital or health system. Duration of disease (acute/chronic, progressive/stable) and time sensitivity of treatment can establish the importance of speed and accuracy in making diagnostic and treatment decisions. Finally, imaging must play a critical role in the diagnosis and management of the patient for a digital diagnostic to make an impact.

Once target indications have been identified, it is important to assess the unmet needs associated with current diagnostic and treatment options, and how imaging contributes to the unmet need. Metrics such as diagnostic accuracy, rate of serious harm resulting from misdiagnosis, and diagnostic invasiveness/risk can elucidate diagnostic unmet needs, whereas availability of current treatment options, treatment invasiveness/risk, and variable treatment outcomes can inform treatment-related unmet needs. For a digital diagnostic to add clinical value, it must be anchored to specific use case(s) that address unmet needs in the patient journey. Providers should consider other diagnostic modalities (e.g., genetic testing, more advanced imaging) as well as non-clinical factors (e.g., patient choice) in terms of their ability to impact the unmet needs or patient management decisions. Shared decision-making is important in areas like oncology, which can sometimes challenge evidence/based medicine.

In many disease states, time is of the essence. In other words, diagnostic findings that are timely and accurate can have a meaningful impact on patient treatment and outcomes (9). In conditions like intracranial hemorrhage, stroke, and pulmonary embolism the ability to quickly diagnose the condition and identify patients eligible for pharmacological and/or surgical intervention can result in reductions in mortality, morbidity, and hospital resource utilization (ICU days, length of hospital stay, etc.).

Likewise, aortic dissection is a rare but acute cardiac event with a very high mortality rate where early diagnosis and surgical intervention is critical to a successful outcome. However, due to clinical presentations that tend to mimic more common problems such as myocardial infarction, vascular embolization, and abdominal conditions, misdiagnosis occurs in up to 30% of cases (10, 11). Despite these issues with improper triage, once patients are referred to CT, sensitivity ranges between 98% and 100% (12). While a digital diagnostic is unlikely to impact the rate of referral to CT, it could expedite time to intervention given the need to mobilize a multidisciplinary team.

Another aspect of time that is important for providers to consider when curating digital solutions is the duration between image acquisition, treatment selection, and outcomes. When the duration is shorter, it is easier to identify the association between the digital output and resulting change in management and patient outcomes. The longer the duration, the more potential exists for confounding factors and other considerations that impact patient management, making it more difficult to measure the impact of a digital diagnostic on patient outcomes.

While curating digital diagnostics, it is important for providers to consider the above disease and related diagnostic features that contribute to the burden of disease. In conditions with poor or variable outcomes where imaging plays a role in patient management, there may be an opportunity for digital diagnostics to provide benefit by establishing unmet needs with current standard of care, defining use cases that inform patient management decisions, and identifying digital solutions that can enhance treatment decision-making.

## Theme 2: do the performance and features of the AI application address the range of unmet needs associated with standard of care?

Once unmet needs have been established, it is important to then evaluate the performance and features of a digital diagnostic in relation to how it influences patient management and outcomes over standard of care.

Key questions to consider:

• When integrated into institutional care pathways, does diagnostic performance provide meaningful resolution vs. standard of care?
• Are there other confounding issues outside of diagnostic performance that influence outcomes (workflow efficiencies, availability of surgical/nursing staff)? Do the solutions in question also address these issues?

Performance within any given use case must be considered with respect to the prevailing gold standard imaging modality, the diagnostic standard of care, and the unmet needs to be addressed. In some circumstances, a digital diagnostic may only need to demonstrate equivalent performance to standard of care, whereas in other cases superior performance over standard of care may be required. When curating digital diagnostics, providers should consider the role of test performance in addressing the underlying unmet needs, which are likely to vary across different use cases.

For example, several digital diagnostics have been developed in oncology, hepatology, and cardiology to provide a non-invasive alternative to diagnostic procedures that are expensive and associated with a range of clinical risks and quality of life implications (13). The ability to replace an invasive biopsy with a non-invasive alternative can expedite time to diagnosis, mitigate risk of exposing patients to unnecessary harms, and optimize resources. For a digital diagnostic to demonstrate the ability to reduce the need for invasive biopsy, the application would need to work with gold-standard imaging modalities upstream of the decision to biopsy and provide comparable accuracy and performance. A few examples include digital diagnostics that quantify coronary obstruction previously undetectable via CT as an alternative to invasive coronary angiography, and similarly, quantification of liver parameters from MR as an alternative to liver biopsy (14, 15).

In oncology, the ability to detect and provide earlier line treatment is often one of the most meaningful drivers of improved outcomes (16, 17). The role of digital diagnostics and their potential to have an impact on outcomes in oncology will depend on the use case, current diagnostic options (including screening tests, diagnostic imaging, and molecular diagnostics), national and institutional evidence-based care pathways, and specific patient sub-populations/risk factors. In breast cancer for example, a variety of screening and supplemental screening imaging techniques (digital mammography, digital breast tomosynthesis, MRI, US, etc.) are recommended based on a woman's age, race/family history, genetic mutations, breast tissue density, and cumulative radiation exposure (18). Performance (sensitivity, false positive rate, etc.) and evidence demonstrating improved net health outcomes varies based on the patient population and screening technique. For a digital diagnostic to provide clinical utility, unmet needs must be clearly defined based on what is contributing to suboptimal outcomes for a given patient population and diagnostic protocol.

Looking across oncology indications can help illustrate how broad the potential range of confounding factors can be when curating digital diagnostics. In the case of intermediate pulmonary nodule risk malignancy stratification, the use of digital diagnostics may assist in both reduction of unnecessary invasive testing for benign patients as well as mitigating delays in treatment for malignant patients (19, 20). In breast cancer, the story is more complex. Improvements in breast cancer detection, particularly in high risk or difficult to diagnose patient populations, can have a meaningful impact on patient management and outcomes if the increase in detection is accompanied by a decrease in number of late-stage cancers. While at the same time, the value of reducing false positives and recall rates is likely to resonate differently, depending on where the institution sits in relation to the 5%–12% recall rate recommended by the ACR (21).

Moreover, while superior accuracy compared to standard of care is desirable where diagnostic performance opportunities exist, equivalence to standard of care may also be acceptable in cases where applications demonstrate the ability to address unmet needs in other ways, especially when considering disease states requiring urgent and/or acute care. For example, digital diagnostics in stroke with comparable performance to standard imaging have demonstrated clinical utility by incorporating mobile notification features that support stroke team coordination and mobilization thereby reducing patient transfer time and time to treatment.

In summary, for a digital diagnostic to address a clinical unmet need it must:

- Integrate into gold standard imaging modalities,
- Target well-defined patient populations with an accepted causal relationship between accurate and timely diagnosis (or risk stratification), treatment decision-making, and patient outcomes
- Be used within reasonable proximity to the decision to conduct further invasive diagnostic testing and/or medical/pharmacologic intervention,
- Align with or influence institutional evidence-based care pathways.

## Theme 3: does the level of documented evidence demonstrate a credible impact on clinical decision-making and patient outcomes?

Once performance vs. standard of care is established, the big question in determining clinical utility is whether the improved diagnostic performance or added features impact patient management and improve outcomes. This is where differences in opinion exist as to whether a change in treatment is sufficient, or whether a digital diagnostic must further demonstrate that net patient outcomes have improved over standard of care. While improving patient outcomes necessitates a change in management, it is important to note that a change in patient management alone does not guarantee an improvement in outcomes. For example, confounding factors including physician discretion in treatment choice, patient adherence, and patient shared decision-making can equally influence management decisions and obscure clinical utility of a digital diagnostic.

The distinction between change in management vs. change in outcomes is important as it is often expensive and time consuming for digital diagnostic developers to conduct large, long-term, patient outcomes studies. While there are exceptions, most third-party payers tend to require evidence of improved outcomes, whether they be safety, efficacy, and/or economic outcomes, whereas providers can exercise more flexibility by inferring outcome benefits or trialing a product for a period of time.

These payor reimbursement and hospital adoption decisions are critical to facilitating continued development of digital diagnostics and realizing their potential for healthcare. In order to balance the payors’ and hospitals’ needs for demonstrated outcomes benefits with fostering innovation and medical advancement through timely access for digital diagnostic development, pathways to reimbursement with evidence development and recognition of shorter-term surrogate endpoints for clinical utility have been and should be considered, especially when those shorter-term endpoints have been shown to have clinical meaningfulness for longer-term outcomes or rare but serious risks. These approaches reduce the early trial investments required to bring digital diagnostics to healthcare, while still providing meaningful short-term evidence, pathways to long-term outcomes evidence, and continued external validation in real-world applications for hospitals, payors, and health systems.

Key questions to consider:

• Is there high-quality evidence demonstrating how the application has influenced patient management decisions?
• Is there high-quality evidence demonstrating how changes in patient management have improved net health outcomes? If not, can improved outcomes be inferred?
• Is the technology endorsed by Guideline committees and HTA authorities?
• How attainable are the benefits within your institution?

When evaluating a digital diagnostic's ability to improve outcomes, it is important to first consider the quality of available evidence. Most of the time, digital diagnostics will receive regulatory clearance on the basis of clinical validation data, often in the form of reader studies, which is informative for establishing performance relative to clinical truth (22). However, validation studies often do not address how a digital diagnostic influences patient management and outcomes in a prospective manner. For providers, the question is what additional evidence is needed to discern whether a digital diagnostic will result in improved net health outcomes. The answer to this question can vary based on the degree of unmet need, ability to infer improved outcomes based on changes in patient management, complexity of care pathways, and real-world experiences from other providers, HTA organizations, and guideline committees.

A robust clinical utility study should be properly controlled with active comparators that reflect the current standard of care, powered to statistically and clinically meaningful outcomes, and compromise patient populations that are representative of the unmet needs. In diseases with high burden (e.g., mortality, morbidity), onboarding digital solutions with lower levels of evidence may be worthwhile tradeoff, particularly if the solution can be evaluated on a trial basis or as part of a clinical program. In other circumstances, it may be appropriate to infer clinical benefit, especially if a digital solution reinforces standard of care (reduces misdiagnosis, supports patient triaging, etc.).

In stroke, the common phrase “time is brain” refers to the criticality of time from symptom to intervention on long-term outcomes. Stroke center accreditation programs require that more than 50% of patients are treated within 60 min of arrival (23). The aforementioned stroke digital diagnostic demonstrated an average reduction of 66 min in transporting the patient from the primary stroke center (PSC) imaging to a comprehensive stroke center (CSC) (24) and an 87 min reduction from time to arrival at CSC to puncture (25). While this same diagnostic eventually demonstrated improved clinical outcomes on commonly accepted stroke outcomes metrics (modified Rankin Scale (mRS) and NIH Stroke Score (NIHSS), the reduction in time to a life-changing/life-saving therapy should be adequate for most hospitals to infer positive net health outcomes benefit, or clinical utility.

In other circumstances, inferring improved outcomes based on evidence demonstrating change in management may not be feasible or clinically appropriate. While fractional flow reserve (FFR)-CT (software for non-invasive quantification of coronary obstruction) demonstrated equivalent diagnostic performance to gold standard FFR as measured during invasive coronary angiography (ICA), performance data alone is inconclusive as to whether FFR-CT obviates the need for ICA, reduces the rate of unnecessary cardiac catheterization, saves money, and most importantly impacts major adverse cardiac events (MACE) or vascular events. As a result, prospective randomized clinical trials powered to long-term clinical outcomes were necessary to demonstrate the clinical utility of FFR-CT, which ultimately demonstrated non-inferior clinical outcomes and superior reductions in ICA and costs (14).

HTA are also a good resource for obtaining a third-party perspective on the concepts described above. Organizations such as AHRQ, NICE, Hayes, and ERCI will publish technology assessments on medical technology. For example, NICE has recently published guidance on the current state of evidence in using AI-CAD software for detecting and measuring lung nodules in CT images (26). In Germany, BfArM has issued a guide for manufacturers, service providers, and users regarding the use of digital health applications (DiGA), introducing the “app on prescription” concept that allows reimbursement of digital applications over a given timeframe, beyond which if evidence targets are not met, reimbursement is cancelled (27). Society guidelines are less likely to be a near-term source of insight as they are typically a trailing indicator of widespread adoption and have been slow to address the use of digital diagnostics.

Lastly, institutions should question how attainable the clinical utility benefits are within their system. Clinical trial data does not always translate to the intricacies of a single hospital or health system due to variability in workflows, population demographics, and technology. This is where real-world data across different hospitals, testimonials, and pilot trials can help with addressing uncertainty. Real-world provider experiences can support confidence that the benefits of the digital diagnostic translate from training and validation data sets into clinical practice. For example, real-world support for digital diagnostics has been used to support the comparative clinical performance of two FDA-approved AI-based computer-aided triage and notification (CADt) detection devices (28) and for the diagnostic performance evaluation of an AI-based CAD for screening mammography (29). For hospitals or health systems considering adopting a digital diagnostic, pilot trials can aid in considering how clinical utility of digital diagnostics may apply within their specific populations and workflows. Pilot trials are small scale test runs over a limited time period aimed at understanding usability of the digital diagnostic before procurement. These studies can provide continued external validation of digital diagnostic performance despite variability in real-world patient populations and hospital or health system practices, both prior to procurement and to monitor performance once implemented.

## Theme 4: what is the practice economic impact of adopting a digital diagnostic?

Digital diagnostics have the potential to provide a range of economic benefits, spanning cost/operational efficiencies through additional revenue generation.

Key questions to consider:

• Will the new technology generate cost-savings or efficiency benefits?
• What is the impact of the application on downstream diagnostic and therapeutic procedures?
• Will the technology improve patient acquisition and retention?
• Is the technology associated with additional reimbursement?

Both workflow efficiency and clinical solutions have demonstrated reductions in radiologist read times, surgery/OR time, length of stay, and associated labor. Some of the cost-saving benefits can be material, such as demonstrating a 3.5-day reduction in neuro-ICU length of stay (24). While operational efficiencies (e.g., staff time) can be difficult to monetize, there is value in reducing burnout and redeploying staff to other patients in need, particularly given nursing and labor shortages. In some cases, these efficiency benefits can be operationalized into revenue enhancing opportunities by optimizing bed space or procedure throughput.

Depending on the use case, digital diagnostics have the potential to impact the entire spectrum of the patient journey, from diagnosis to treatment. For digital diagnostics that improve diagnosis, health systems may see improvements in downstream revenues associated with additional diagnostic procedures, surgery, radiation, and biologic therapy. Depending on the unmet need being addressed, digital diagnostics may also have the potential to improve patient acquisition and retention rates.

Applications that have demonstrated clinical utility also have the potential of generating economic benefits through higher quality patient management, including reduced rehospitalizations and need for follow-up care. With continued transition to value-based care, digital diagnostics can play a role in impacting quality measures, outcomes-based agreements, and patient satisfaction.

## Conclusion

There is an opportunity for digital diagnostics to transform healthcare delivery. As the number of digital diagnostics entering the market continues to grow, not all of them will provide the same level of benefit. Providers need to be clear in what they hope to achieve with digital diagnostics, as the benefits span workflow efficiencies and patient outcomes. If improving patient outcomes is the goal, this paper suggests 4 guiding themes for curating digital diagnostics on the basis of clinical utility (Table 2).

**Table 2:** Guiding themes for healthcare providers to consider when curating digital diagnostics based on clinical utility.

Understand the unmet needs that underpin the clinical burden of the disease	Evaluate performance and solution features vs. standard of care	Critique level of evidence demonstrating impact on patient management and outcomes	Practice economic implications
• Clinical burden of disease across key outcome domains • Impact of current diagnostics and treatments on patient outcomes • Role of imaging in treatment decision-making • Duration of time between imaging, treatment selection, and patient outcomes	• Use case codification throughout the patient journey • Diagnostic performance vs. standard of care • Influence of additional product features on unmet needs	• Quality and rigor of the supporting evidence base • Frequency with which the solution influences patient management decisions • Association between changes in patient management and improved net health outcomes • Level of outside endorsement • Attainability of benefits within your institution	• Cost-savings or efficiency benefits • Impact on downstream procedure revenue • Patient acquisition and retention benefits • Availability of additional reimbursement

## Data Availability

The original contributions presented in the study are included in the article/Supplementary Material, further inquiries can be directed to the corresponding author.

## References

[1] Hosny A Parmar C Quackenbush J Schwartz LH Aerts HJWL. Artificial intelligence in radiology. Nat Rev Cancer. (2018) 18(8):500–10. 10.1038/s41568-018-0016-5, 29777175 PMC6268174

[2] Makary MS Vitellas CA. Healthmanagement.org. J. (2021) 21:205–8.

[3] Seah J Boeken T Sapoval M Goh GS. Prime time for artificial intelligence in interventional radiology. Cardiovasc Intervent Radiol. (2022) 45(3):283–9. 10.1007/s00270-021-03044-4, 35031822 PMC8921296

[4] Benjamens S Dhunnoo P Meskó B. The state of artificial intelligence-based FDA-approved medical devices and algorithms: an online database. npj Digit. Med. (2020) 3:118. 10.1038/s41746-020-00324-0 32984550 PMC7486909

[5] Institute ACoRsDS. FDA Cleared AI-Algorithms. (2022). Available online at: https://aicentral.acrdsi.org/ (accessed November 2022)

[6] Center RUM. AI for Radiology. (2022). Available online at: https://grand-challenge.org/aiforradiology/ (accessed November 2022)

[7] Davenport TH Glaser JP. Factors governing the adoption of artificial intelligence in healthcare providers. Discov Health Syst. (2022) 1(1):4. 10.1007/s44250-022-00004-8 37521111 PMC9628307

[8] Lobig F Subramanian D Blankenburg M Sharma A Variyar A Butler O. To pay or not to pay for artificial intelligence applications in radiology. npj Digit Med. (2023) 6:117. 10.1038/s41746-023-00861-4 37353531 PMC10290087

[9] Holmboe ES Durning SJ. Assessing clinical reasoning: moving from in vitro to in vivo. Diagnosis (Berl). (2014) 1(1):111–7. 10.1515/dx-2013-0029 29539977

[10] Levy D Goyal A Grigorova Y Farci F Le JK. Aortic dissection. In Statpearls. Treasure Island, FL: StatPearls Publishing (2023). Available online at: https://www.ncbi.nlm.nih.gov/books/NBK441963/ (updated April 23, 2023) 28722992

[11] Huo D Kou B Zhou Z Lv M. A machine learning model to classify aortic dissection patients in the early diagnosis phase. Sci Rep. (2019) 9:2701. 10.1038/s41598-019-39066-9 30804372 PMC6389887

[12] Vardhanabhuti V Nicol E Morgan-Hughes G Roobottom CA Roditi G Hamilton MC Recommendations for accurate CT diagnosis of suspected acute aortic syndrome (AAS)–on behalf of the British society of cardiovascular imaging (BSCI)/British society of cardiovascular CT (BSCCT). Br J Radiol. (2016) 89(1061):20150705. 10.1259/bjr.20150705 26916280 PMC4985448

[13] Neuberger J Cain O. The need for alternatives to liver biopsies: non-invasive analytics and diagnostics. Hepat Med. (2021) 14(13):59–69. 10.2147/HMER.S278076 PMC8214024 34163263

[14] Douglas PS De Bruyne B Pontone G Patel MR Norgaard BL Byrne RA 1-year outcomes of FFRCT-guided care in patients with suspected coronary disease: the PLATFORM study. J Am Coll Cardiol. (2016) 68(5):435–45. 10.1016/j.jacc.2016.05.057 27470449

[15] Andersson A Kelly M Imajo K Nakajima A Fallowfield JA Hirschfield G Clinical utility of magnetic resonance imaging biomarkers for identifying nonalcoholic steatohepatitis patients at high risk of progression: a multicenter pooled data and meta-analysis. Clin Gastroenterol Hepatol. (2022) 20(11):2451–2461.e3. 10.1016/j.cgh.2021.09.041 34626833

[16] Crosby D Bhatia S Brindle KM Coussens LM Dive C Emberton M Early detection of cancer. Science. (2022) 375(6586):eaay9040. 10.1126/science.aay9040 35298272

[17] Whitaker K. Earlier diagnosis: the importance of cancer symptoms. Lancet Oncol. (2020) 21(1):6–8. 10.1016/S1470-2045(19)30658-8 31704136

[18] Monticciolo DL Newell MS Moy L Lee CS Destounis SV. Breast cancer screening for women at higher-than-average risk: updated recommendations from the ACR. J Am Coll Radiol. (2023) S1546–1440(23):00334–4. 10.1016/j.jacr.2023.04.002 37150275

[19] Massion PP Antic S Ather S Arteta C Brabec J Chen H Assessing the accuracy of a deep learning method to risk stratify indeterminate pulmonary nodules. Am J Respir Crit Care Med. (2020) 202(2):241–9. 10.1164/rccm.201903-0505OC 32326730 PMC7365375

[20] Baldwin DR Gustafson J Pickup L Arteta C Novotny P Declerck J External validation of a convolutional neural network artificial intelligence tool to predict malignancy in pulmonary nodules. Thorax. (2020) 75(4):306–12. 10.1136/thoraxjnl-2019-214104 32139611 PMC7231457

[21] Lehman CD Arao RF Sprague BL Lee JM Buist DS Kerlikowske K National performance benchmarks for modern screening digital mammography: update from the breast cancer surveillance consortium. Radiology. (2017) 283(1):49–58. 10.1148/radiol.2016161174 27918707 PMC5375631

[22] Khunte M Chae A Wang R Jain R Sun Y Sollee JR Trends in clinical validation and usage of US food and drug administration-cleared artificial intelligence algorithms for medical imaging. Clin Radiol. (2023) 78(2):123–9. 10.1016/j.crad.2022.09.122 36625218

[23] American heart association & American stroke association “get with the guidelines—stroke recognition” hospital recognition criteria 2023–2024, get with the guidelines®—stroke recognition criteria. American Heart Association (AHA) (2023). Available online at: https://www.heart.org/en/professional/quality-improvement/get-with-the-guidelines/get-with-the-guidelines-stroke/get-with-the-guidelines-stroke-recognition-criteria (Accessed September 2023).

[24] Hassan AE Ringheanu VM Rabah RR Preston L Tekle WG Qureshi AI. Early experience utilizing artificial intelligence shows significant reduction in transfer times and length of stay in a hub and spoke model. Interv Neuroradiol. (2020) 26(5):615–22. 10.1177/1591019920953055 32847449 PMC7645178

[25] Hassan AE Ringheanu VM Preston L Tekle WG. Artificial intelligence–parallel stroke workflow tool improves reperfusion rates and door-in to puncture interval. Stroke Vasc Interv Neurol. (2022) 2:e000224. 10.1161/SVIN.121.000224 PMC12778824 41584017

[26] National Institute for Health and Care Excellence (NICE). AI-derived Computer-Aided Detection (CAD) Software for Detecting and Measuring Lung Nodules in CT Scan Images [Diagnostics Guidance [DG55]], Overview | AI-derived Computer-Aided Detection (CAD) Software for Detecting and Measuring Lung Nodules in CT Scan Images | Guidance | NICE. (2023). (accessed September 2023)

[27] Federal Institute for Drugs and Medical Devices. The fast-track process for digital health applications (DiGA) according to section 139e SGB V. A guide for manufacturers, service providers and users (Version 1.0). BfArM—Digit Health Appl (DiGA). (2020).

[28] Kunst M Gupta R Coombs LP Delfino JG Khan A Berglar I Real-world performance of large vessel occlusion artificial intelligence-based computer-aided triage and notification algorithms-what the stroke team needs to know. J Am Coll Radiol. (2023) 21(2):329–40. 10.1016/j.jacr.2023.04.0032024 37196818

[29] Lee SE Hong H Kim EK. Diagnostic performance with and without artificial intelligence assistance in real-world screening mammography. Eur J Radiol Open. (2024) 12:100545. 10.1016/j.ejro.2023.100545 38293282 PMC10825593

